# Dose-Response Reduction in Risk of Nasopharyngeal Carcinoma From Smoking Cessation: A Multicenter Case-Control Study in Hong Kong, China

**DOI:** 10.3389/fonc.2021.699241

**Published:** 2021-09-27

**Authors:** Lijun Wang, Zhi-Ming Mai, Roger Kai-Cheong Ngan, Wai-Tong Ng, Jia-Huang Lin, Dora Lai-Wan Kwong, Shing-Chun Chiang, Kam-Tong Yuen, Alice Wan-Ying Ng, Dennis Kai-Ming Ip, Yap-Hang Chan, Anne Wing-Mui Lee, Maria Li Lung, Sai Yin Ho, Tai-Hing Lam

**Affiliations:** ^1^ School of Public Health, University of Hong Kong, Hong Kong, China; ^2^ Centre for Nasopharyngeal Carcinoma Research (CNPCR), Research Grants Council Area of Excellence Scheme, the University of Hong Kong, Hong Kong, China; ^3^ Radiation Epidemiology Branch, Division of Cancer Epidemiology and Genetics, National Cancer Institute, Bethesda, MD, United States; ^4^ Department of Clinical Oncology, Queen Elizabeth Hospital, Hong Kong, China; ^5^ Department of Clinical Oncology, the University of Hong Kong, Hong Kong, China; ^6^ Department of Epidemiology and Biostatistics, School of Public Health, Peking University Health Science Centre, Beijing, China; ^7^ Department of Clinical Oncology, Queen Mary Hospital, The University of Hong Kong, Hong Kong, China; ^8^ Department of Clinical Oncology, Princess Margaret Hospital, Hong Kong, China; ^9^ Department of Clinical Oncology, Tuen Mun Hospital, Hong Kong, China; ^10^ Department of Medicine, Queen Mary Hospital, the University of Hong Kong, Hong Kong, China

**Keywords:** nasopharyngeal carcinoma, cigarette smoking, smoking cessation, case-control study, dose-response relation

## Abstract

**Background:**

Cigarette smoking is associated with nasopharyngeal cancer (NPC) risk. Whether quitting reduces the risk is unclear. We investigated the associations of NPC with duration of and age at quitting in an endemic region.

**Methods:**

We investigated the associations between NPC and quitting in a multicenter case-control study in Hong Kong with 676 newly diagnosed NPC cases and 1,285 hospital controls between 2014 and 2017, using a computer-assisted self-administered questionnaire. Multivariable unconditional logistic regression yielded adjusted odds ratios (AORs) of NPC by quitting status, duration and age of quitting, combinations of duration and age of quitting, and quitting to smoking duration ratio, compared with current smoking.

**Results:**

Quitting (AOR: 0.72; 95% CI: 0.53–0.98) and never smoking (0.73, 0.56–0.95) were associated with lower NPC risk. NPC risk decreased with (i) longer quitting duration (*p* < 0.01), reaching significance after 11–20 (0.62, 0.39–0.99) and 21+ years (0.54, 0.31–0.92) of quitting; (ii) younger quitting age (*p* = 0.01), reaching significance for quitting at <25 years (0.49, 0.24–0.97); and (iii) higher quitting to smoking duration ratio (*p* < 0.01), reaching significance when the ratio reached 1 (0.60, 0.39–0.93). Quitting younger (age <25) appeared to confer larger reductions (49% for ≤10 years of quitting, 50% for 11+ years) in NPC risk than quitting at older ages (25+) regardless of quitting duration (16% for ≤10 years, 39% for 11+ years).

**Conclusions:**

We have shown longer duration and younger age of quitting were associated with lower NPC risk, with dose-response relations. Our findings support including smoking as a cause of NPC. Stronger tobacco control measures and quitting services are needed to prevent NPC.

## Introduction

Despite its similar cellular or tissue lineage, nasopharyngeal carcinoma (NPC) shows a unique epidemiologic pattern among head and neck cancers. Nonkeratinizing undifferentiated NPC (World Health Organization [WHO] type III) is the predominant histology subtype in East and Southeast Asia, where 71% of NPC cases worldwide occurred, while squamous cell carcinoma and keratinizing undifferentiated carcinoma (WHO type I and II) are predominant in nonendemic regions (1–3). The incidence of NPC also peaks at an earlier age in endemic (45–54 years) than nonendemic (65–74 years) regions (4, 5).

Multiple factors could contribute to the development of NPC, such as Epstein-Barr virus (EBV) infection, host genetics, family history, and occupational exposure to dusts and formaldehyde (1, 6). Asian studies in endemic regions also reported the associations with early life consumption of salted fish (7–9). In 2004 and 2012, the International Agency for Research on Cancer reported that cigarette smoking increases NPC risk in both endemic and nonendemic regions (10, 11), while the Health Consequences of Smoking Report by the US Surgeon General in 2014 only concluded on the carcinogenicity for oropharynx but not for nasopharynx due to insufficient evidence (12). After that, further evidence has emerged (1, 13–16) and a meta-analysis showed that every 10 more pack-years of smoking was associated with 15% higher risk of NPC (17). Our recent individual data meta-analysis of cohort studies in endemic regions has also provided strong evidence for a causal relation between smoking and NPC (18). In contrast, the relation between quitting smoking and NPC remains unclear, and the latest 2020 report of the US Surgeon General on smoking cessation has not stated that quitting reduced the risk of NPC (19). Studies that directly compared NPC risks between former and current smoking were few (13, 20, 21), and the adjusted odds ratio (AOR) in quitters (vs. never smokers) appeared to be even higher than that in current smokers (vs. never smokers) in some studies (22, 23).

A case-control study in the USA, a nonendemic region, reported that compared with current smokers, the risks of squamous cell NPC were lower in 32 quitters who had stopped smoking for less than 5 years (AOR: 0.1; 95% confidence interval (CI): 0.0–0.6), 5–14 years (0.2, 0.1–0.7), and 15+ years (0.2, 0.0–0.8) (20). Although the *p* for trend was significant (*p* for trend = 0.003), the AORs did not support a dose-response relation. Four case-control studies have reported the associations between duration of quitting and NPC in endemic regions. One, including 681 NPC cases and 1,078 controls in Thailand, showed a lower NPC risk in quitters who had stopped smoking for >5 years (AOR: 0.65; 95% CI: 0.45–0.96) compared with current smokers, with WHO type III NPC accounting for 68.4% of all cases (21). Another case-control study in Queen Elizabeth Hospital of Hong Kong showed a suggestive lower risk of NPC in quitters who had stopped smoking for 10+ years (0.79, 0.43–1.45), but the study had a small sample size of 73 former smokers among 352 cases (93.2% were WHO type III) (13). Another large case-control study (1,857 NPC cases and 1,907 community controls) in Guangdong and Guangxi, southern China showed that compared with never smokers, NPC risk was lower for short-term quitters (quitting for ≤9 years; AOR: 0.54; 95% CI: 0.39–0.75) but higher for long-term quitters (≥10 years; 1.44, 1.04–2.00) (15). The latest case-control study in Taiwan reported that, compared with never smoking, quitting for ≤5 years was associated with a higher NPC risk (1.54, 1.04–2.29), and quitting for ≥6 years showed no association (1.02, 0.67–1.56 for 6–10 years; 1.25, 0.93–1.69 for >10 years) (16). We have found no reports on NPC risk by age at quitting and smoking duration as of July 2021.

In Hong Kong with one of the highest NPC incidence rates in the world and over 90% cases being nonkeratinizing NPC, we investigated the associations between quitting smoking and NPC risk, using data from the Hong Kong Area of Excellence NPC (HKAoENPC) study with detailed information on quitting and potential confounders.

## Methods

### Study Subjects

The HKAoENPC study is a multicenter case-control study with fieldwork conducted between March 2014 and September 2017 in five major regional hospitals (Queen Mary Hospital, Pamela Youde Nethersole Eastern Hospital, Queen Elizabeth Hospital, Princess Margaret Hospital, and Tuen Mun Hospital) that treated up to 75% of all new NPC cases in Hong Kong. Details of the methods have been reported (24, 25).

Briefly, the cases were primary NPC patients diagnosed with histological and/or radiological evidence within 2 months. The controls were 1,502 frequency-matched (by sex and 5-year age group) patients or referrals with a new health complaint in the past 12 months in specialist outpatient clinics or new inpatients admitted in the past 3 months in the same hospitals. Those with a history of NPC, dementia, or suspected NPC symptoms such as recent unilateral facial nerve palsy, tinnitus, unilateral hearing loss, and epistaxis were excluded. Following the AsiaLymph guideline of the US National Cancer Institute, we specified that no more than 15% of controls would have the same disease. Controls in specific conditions were further excluded based on known or suspected risk factors of NPC, including immunological, infectious, and/or inflammatory etiology. In the present analysis, we excluded 297 (12.8%) subjects aged over 65 to reduce reverse causality, as smoking cessation in older people was usually due to illness (the “sick quitter” effect) (26, 27). Another 59 (2.5%) subjects were excluded due to missing information on smoking. In total, 1,961 (676 NPC cases and 1,285 hospital controls) were included.

The Institutional Review Board of the HKU/Hospital Authority HK West Cluster (UW 11-192), the HK East Cluster Research Ethics Committee (HKEC-2012-043), the Research Ethics Committee of the Hospital Authority Kowloon Central/Kowloon East (KC/KE-13-0115/ER-2), the Research Ethics Committee of the Kowloon West Cluster [KW/EX-13-073(63–11)], and the NTW Cluster Clinical & Research Ethics Committee (NTWC/CREC/1239-13) approved the study. Written informed consent was obtained from all subjects.

### Assessment of Smoking, Quitting, and Covariates

We used a computer-assisted, self-administered questionnaire to minimize interviewer bias. Smoking was assessed by “Have you ever smoked (at least 1 cigarette per day for 6 consecutive months)?” with options of “never smoking,” “current smoking,” “former smoking,” and “don’t know.” Those who chose “former smoking” answered either “How many years have you quit smoking?” or “At what age did you stop smoking?.” Both duration of quitting and age at quitting were assessed based on the answered question. Subjects who chose “current smoking” or “quitting for less than 2 years” were classified as current smokers, and those who had quit for 2 years or longer were classified as former smokers. The duration of quitting (2–4, 5–10, 11–20, and 21+ years) and age at quitting (45+, 35–44, 25–34, and <25 years) were each classified into four groups. We asked “how many cigarettes do you usually smoke per day” in subjects who still smoked and the numbers of cigarettes smoked per day before quitting in those who had quit. The duration of smoking was calculated by subtracting the age at smoking initiation (“At what age did you start smoking?”) from the age at quitting. The duration of quitting was divided by the duration of smoking to calculate the quitting to smoking duration ratio and recoded as <0.5, 0.5–<1, and ≥1. We hypothesized that longer duration of quitting relative to smoking (larger values of quitting to smoking duration ratio) was associated with lower NPC risk. We reinterviewed 140 subjects after around 30 weeks and found high reliability coefficients (0.84–1.00) of smoking and quitting status (25).

Information on sociodemographic characteristics, lifestyle, and putative risk factors of NPC was collected, including sex, age, education, parental education, housing type at the age of 10 years, current household income, alcohol drinking (at least once a week for 6 consecutive months), consumption of salted fish at 6–12 years old (yes/no), family history of cancer (none; non-NPC cancers; NPC), and exposure to occupational hazards (including dust, chemical gas, fumes, acid, or alkali) (yes/no).

### Statistical Analysis

Chi-square test was used to assess the differences in background characteristics between cases and controls. To analyze the associations of NPC with smoking status, duration of quitting and age at quitting, odds ratios (ORs) and 95% CIs were calculated using unconditional multivariable logistic regression models, adjusting for potential confounders including sex, age, education, parental highest education, housing type at the age of 10 years, current household income, alcohol drinking, and the number of cigarettes smoked per day. Despite being a strong risk factor for NPC, EBV was not adjusted for as it has been found to mediate the association between smoking and NPC (16, 28, 29). To assess dose-response relation, we tested the linear trends of NPC risk by the duration of quitting, age at quitting, and quitting to smoking duration ratio. Given the high negative correlation between duration of quitting and age at quitting, we assessed NPC risks for different combinations of quitting duration and age at quitting, rather than mutually adjusting for them in regression models.

As sex, consumption of salted fish at age 6–12 years, family history of cancer, and exposure to occupational hazards are putative risk factors of NPC (6–9) and could modify the effect of quitting smoking on NPC, interaction effects were assessed by introducing the interaction (cross-product) terms into regression models. All statistical analyses were conducted using R version 4.0.0 (R Foundation for Statistical Computing Platform). All tests were two-sided with *p* < 0.05 indicating statistical significance.

## Results

Compared with 1,285 controls, the 676 NPC cases had lower education levels (self and parental) and current household income, but higher proportions of family history of NPC, alcohol drinking, and exposure to occupational hazards (all *p*-values <0.05). No difference was observed for housing type at the age of 10 years, consumption of salted fish at age 6–12, and number of cigarettes smoked per day in ever smokers (**Table 1**).

**Table 1:** Characteristics of nasopharyngeal carcinoma cases and controls in subjects aged 18–65.

	Cases (*n* = 676)		Controls (*n* = 1,285)		*p* ^a^
	*n*	%	*n*	%	
Sex (men)	495	73.2	864	67.2	0.01
Age at diagnosis/referral (years)					
Mean (SD)	50.1 (9.7)	–	48.8 (11.4)	–	
18–39	113	16.7	275	21.4	0.045
40–54	295	43.6	522	40.6	
55–65	268	39.6	488	38.0	
Education					<0.001
Primary or below	123	18.2	171	13.3	
Secondary	435	64.3	729	56.8	
Tertiary	118	17.5	384	29.9	
Parental highest education					<0.001
Primary or below	406	71.2	705	63.0	
Secondary	149	26.1	335	29.9	
Tertiary	15	2.6	79	7.1	
Housing type at the age of 10 years					0.29
Owned	197	29.3	405	31.7	
Temporary, rent, or others	476	70.7	873	68.3	
Current household income (HK$/month^b^)					<0.001
≤14,999	160	24.8	292	24.4	
15,000–24,999	217	33.6	316	26.5	
25,000–39,999	140	21.7	248	20.8	
40,000+	129	20.0	338	28.3	
Alcohol drinking	294	48.3	508	41.6	0.01
Salted fish consumption at aged 6–12	556	82.4	1,019	79.5	0.14
Family history of cancer					<0.001
None	244	40.4	631	52.1	
Non-NPC cancers	241	39.9	510	42.1	
NPC	119	19.7	69	5.7	
Occupational hazards	342	56.2	503	41.3	<0.001
Average number (SD) of cigarettes smoked per day in ever smokers	16.4	9.3	15.6	10.1	0.32
NPC histology [% (case only)]					
WHO type I	12	1.8	–	–	
WHO type II	16	2.4	–	–	
WHO type III	641	94.8	–	–	
Unknown	7	1.0	–	–	

Missing rates were 13.9% for parental highest education, 6.2% for current household income, 6.7% for alcohol drinking status, 7.5% for family cancer history, 6.8% for occupational hazards, 3.7% for the number of cigarettes smoked per day, and less than 1% for other variables.

a *P*-values of differences in characteristics between case and control groups, using x^2^ test..

b US$1 = HK$7.8.

**Table 2** shows that compared with current smoking, both quitting (AOR: 0.72; 95% CI: 0.53–0.98) and never smoking (0.73, 0.56–0.95) were associated with lower risks of NPC. The ORs of NPC generally decreased with increasing duration of quitting (*p* for trend: <0.01), reaching statistical significance after quitting for 11–20 (AOR: 0.62; 95% CI: 0.39–0.99) and 21+ years (0.54, 0.31–0.92). NPC risks were lower for quitting at younger age (*p* for trend = 0.01), reaching significance at age <25 (0.49, 0.24–0.97) but were not significant for quitting at age 25–34 (0.68, 0.42–1.11), 35–44 (0.72, 0.43–1.23), and 45+ (0.73, 0.44–1.19). The AORs showed dose-response relations for duration of quitting and age at quitting. Similar patterns of associations and trends were observed in men, but the results were unstable in women due to small number of quitters.

**Table 2:** Associations between quitting smoking and nasopharyngeal carcinoma (cases *vs*. controls) in subjects aged 18–65 by sex.

	All^a^ (*n* = 1,961)			Men (*n* = 1,359)			Women (*n* = 602)		
	Cases/controls	Sex and age adjusted (95% CI)^b^	Multivariable-adjusted OR (95% CI)^c^	Cases/controls	Sex and age adjusted (95% CI)^b^	Multivariable-adjusted OR (95% CI)^c^	Cases/controls	Sex and age adjusted (95% CI)^b^	Multivariable-adjusted OR (95% CI)^c^
Smoking status^d^									
Current	194/257	Ref	Ref	176/231	Ref	Ref	18/26	Ref	Ref
Former	108/202	0.67 (0.49, 0.90)**	0.72 (0.53, 0.98)*	101/182	0.68 (0.49, 0.93)*	0.74 (0.53, 1.02)	7/20	0.50 (0.18, 1.44)	0.54 (0.18, 1.63)
Never	374/826	0.62 (0.49, 0.78)***	0.73 (0.56, 0.95)*	218/451	0.62 (0.48, 0.81)***	0.73 (0.54, 0.99)*	156/375	0.58 (0.30, 1.10)	0.71 (0.31, 1.64)
Duration of quitting (years)									
2–4	20/26	0.97 (0.52, 1.80)	1.02 (0.54, 1.90)	19/23	1.02 (0.54, 1.95)	1.08 (0.56, 2.08)	1/3	– (–)	– (–)
5–10	28/55	0.65 (0.40, 1.07)	0.71 (0.43, 1.19)	23/47	0.61 (0.36, 1.05)	0.67 (0.38, 1.16)	5/8	0.90 (0.24, 3.39)	0.76 (0.17, 3.38)
11–20	33/66	0.59 (0.37, 0.94)*	0.62 (0.39, 0.99)*	33/59	0.66 (0.41, 1.07)	0.70 (0.43, 1.13)	0/7	– (–)	– (–)
21+	27/55	0.51 (0.30, 0.86)*	0.54 (0.31, 0.92)*	26/53	0.53 (0.31, 0.89)*	0.55 (0.32, 0.96)*	1/2	– (–)	– (–)
* P for trend^e^*		0.001	<0.01		<0.01	0.01		-	-
Age at quitting									
45+	37/57	0.67 (0.41, 1.09)	0.73 (0.44, 1.19)	36/56	0.69 (0.42, 1.12)	0.74 (0.45, 1.22)	1/1	– (–)	– (–)
35–44	26/46	0.69 (0.41, 1.17)	0.72 (0.43, 1.23)	24/43	0.69 (0.40, 1.19)	0.73 (0.42, 1.26)	2/3	0.75 (0.10, 5.43)	0.73 (0.07, 7.16)
25–34	32/63	0.65 (0.41, 1.04)	0.68 (0.42, 1.11)	30/53	0.71 (0.43, 1.16)	0.76 (0.46, 1.27)	2/10	0.31 (0.06, 1.65)	0.23 (0.04, 1.41)
<25	13/36	0.48 (0.25, 0.93)*	0.49 (0.24, 0.97)*	11/30	0.47 (0.23, 0.97)*	0.49 (0.23, 1.04)	2/6	0.51 (0.09, 2.98)	0.51 (0.07, 3.98)
* P for trend^e^*		<0.01	0.01		0.01	0.04		0.17	0.17

*p < 0.05, **p < 0.01, ***p < 0.001. OR, odds ratio; CI, confidence interval.

ORs were not shown due to insufficient subjects (<2 NPC cases) in the corresponding categories.

a No interaction with sex, salted fish consumption, family history of cancer, or exposure to occupational hazards (p-values for interaction ranging from 0.14 to 0.99).

b Adjusted for sex and 5-year age group.

c Adjusted for sex, 5-year age group, education, parental highest education, housing type at the age of 10 years, current household income, alcohol drinking, and the number of cigarettes smoked per day.

d Current smokers included ever smokers who quit for <2 years. Quitting was defined as having stopped smoking for ≥2 years.

e p-values for trends with current smokers as the reference group.

**Table 3** shows that quitting younger at age <25 appeared to confer larger reductions in NPC risk after quitting for both ≤10 years (AOR: 0.51; 95% CI: 0.10–2.55) and 11+ years (0.50, 0.24–1.05) than quitting at age ≥25 after quitting for ≤10 years (0.84, 0.54–1.29) and 11+ years (0.61, 0.40–0.93). **Table 4** shows dose-response relation that NPC risk decreased with quitting to smoking duration ratio (*p* for trend: <0.01), reaching statistical significance when the quitting duration was as long as the smoking duration (ratio ≥1; 0.60, 0.39–0.93).

**Table 3:** Nasopharyngeal carcinoma risk for combinations of quitting duration and age at quitting in subjects aged 18–65.

	Cases/controls	Sex- and age-adjusted OR (95% CI)^a^	Multivariable-adjusted OR (95% CI)^b^
Current smoking^c^	194/257	Ref	Ref
Quitting older + shorter duration	46/73	0.78 (0.51, 1.19)	0.84 (0.54, 1.29)
Quitting older + longer duration	49/93	0.58 (0.38, 0.88)*	0.61 (0.40, 0.93)*
Quitting younger + shorter duration	2/8	0.49 (0.10, 2.42)	0.51 (0.10, 2.55)
Quitting younger + longer duration	11/28	0.47 (0.23, 0.98)*	0.50 (0.24, 1.05)

*p < 0.05. OR, odds ratio; CI, confidence interval. Notes: Age at quitting was classified as <25 and 25+ years, and duration of quitting was classified as ≤10 and 11+ years.

a Adjusted for sex and 5-year age group.

b Adjusted for sex, 5-year age group, education, parental highest education, housing type at the age of 10 years, current household income, alcohol drinking, and the number of cigarettes smoked per day. No interaction with sex, salted fish consumption, family history of cancer, or exposure to occupational hazards (p-values for interaction ranging from 0.15 to 0.99).

c Current smokers included ever smokers who quit for <2 years. Quitting was defined as having stopped smoking for ≥2 years.

**Table 4:** Nasopharyngeal carcinoma risk by quitting to smoking duration ratio in subjects aged 18–65.

	Cases/controls	Sex- and age-adjusted OR (95% CI)^a^	Multivariable-adjusted OR (95% CI)^b^
Current smoking^c^	194/257	Ref	Ref
Quitting to smoking duration ratio <0.5	48/76	0.74 (0.49, 1.12)	0.78 (0.51, 1.19)
Quitting to smoking duration ratio 0.5–<1	18/36	0.62 (0.34, 1.13)	0.65 (0.35, 1.19)
Quitting to smoking duration ratio ≥1	41/87	0.56 (0.37, 0.86)**	0.60 (0.39, 0.93)*
P for trend		<0.01	<0.01

*p < 0.05, **p < 0.01. OR, odds ratio; CI, confidence interval.

a Adjusted for sex and 5-year age group.

b Adjusted for sex, 5-year age group, education, parental highest education, housing type at the age of 10 years, current household income, alcohol drinking, and the number of cigarettes smoked per day. No interaction with sex, salted fish consumption, family history of cancer, or exposure to occupational hazards (p-values for interaction ranging from 0.33 to 0.83).

c Current smokers included ever smokers who quit for <2 years. Quitting was defined as having stopped smoking for ≥2 years.

None of the putative risk factors, including sex, consumption of salted fish at age 6–12 years, family history of cancer, and exposure to occupational hazards, showed interaction with quitting status, duration of quitting, or age at quitting in the associations.

## Discussion

This study showed dose-response reductions in risk of nasopharyngeal carcinoma with younger age at quitting and longer duration of quitting. NPC risk reduction was 29% for 5–10 years of cessation, 38% for 11–20 years, and 46% for 21+ years. NPC risk was halved by quitting smoking before the age of 25 years. These patterns were consistent with those for other types of head and neck cancers (30, 31). We did not find effect modification by the three major putative risk factors of NPC—consumption of salted fish at 6–12 years, family history of cancer, and exposure to occupational hazards—in the associations between quitting smoking and NPC.

In line with “reversibility” in Bradford Hill criteria for causality, our findings that quitting smoking was associated with reduced NPC risk strengthen the existing relation between smoking and NPC (13–16, 20, 21, 23, 31–41). We found a nonsignificantly lower risk after quitting for 5 to 10 years and significantly lower risks after 11 years. Risk reduction of NPC by age at quitting has not been reported previously. Although NPC risk declined with quitting at younger age, only quitting before 25 years was associated with a significantly reduced risk.

Despite that both age and duration of quitting were associated with NPC risk, smokers should not delay quitting and try to compensate by a longer duration of quitting, because we also found that quitting at young age could be more important than quitting for a long duration. In former smokers who quit younger than 25, NPC risk was halved regardless of quitting duration even for ≤10 years, but in those who quit at or older than 25, risk reduction appeared to be smaller even after quitting for 11+ years (38%). We first used quitting to smoking duration ratio to test the dose-response effects of quitting duration accounting for the effects of smoking duration, which could also be applicable to other health outcomes. The results showed that NPC risk decreased with higher quitting to smoking duration ratio and was reduced by 40% when the ratio was ≥1, i.e., quitting duration reached smoking duration. Quitting young means lower cumulative exposure to tobacco smoke and less harm on health that may take shorter time to reverse (42). This “early quitting protective effect” is plausible because of the young peak age of NPC in endemic regions such as Hong Kong (4, 5). Why such results were observed in Hong Kong but not elsewhere can also be explained by its large decrease in smoking prevalence with more quitters in general and in NPC patients than in China Mainland and other endemic regions (43).

As with previous studies (14, 44), the associations were mainly observed in men but not in women, probably due to the small numbers of NPC and smokers in women. Our individual data meta-analysis of cohort studies on 334,935 men in endemic regions only observed 48 NPC cases who had quit cigarette smoking, and the risk reduction was nonsignificant (22 cases who quit for <5 years—AOR: 1.22; 95% CI: 0.78–1.90; 26 cases who had quit for ≥5 years: 0.91, 0.60–1.36) (18). The above results suggest that case-control study is probably the most feasible study type for causal inference between quitting smoking and NPC, especially when assessing dose-response relations.

The incidence of NPC increases with age and peaks at 45–59 years in Hong Kong and other high-risk regions, and a much earlier subpeak appears at 15–19 years in low-risk regions (5). The peak age is younger than those of other solid tumors, and thus quitting smoking as young as possible is recommended for preventing NPC. Our ecological study has shown concurrent declining trends of smoking and NPC prevalence in past decades in Hong Kong (45), suggesting that increasing tobacco tax, and/or other tobacco control measures, could contribute to NPC prevention.

Previous NPC studies defined current smoking in periods ranging from past month to past year and showed no reduction or even an increase in NPC risk for quitters than current smoking (13, 15, 21–23, 46). For example, a case-control study with 113 NPC cases and 1,910 controls reported an AOR (95% CI) of 2.3 (1.3–4.0) for former smoking and 1.4 (0.8–2.6) for current smoking vs. never smoking (22). The most plausible explanation is reverse causality that quitting was driven by illness or ascertainment of NPC. Therefore, we classified recent quitting as current smoking, in line with other smoking-related epidemiological studies (13, 47, 48). On the other hand, if the recent quitters had higher NPC risk, classifying them as current smokers would diminish the differences in NPC risk between the remaining former smokers and never smokers. Compared with never smokers, the smaller AORs in former smokers who quit for over 11 years or before 25 years old should not be interpreted as protective effects of former smoking, considering the overlapping CIs. Further studies are warranted to investigate the potential drivers, such as adoption of other health behaviors (e.g., diet, physical activity) among those who quit smoking early.

Given that the excess NPC risk from smoking was partially mediated through EBV reactivation (16, 29, 49), quitting smoking would incur additional benefits apart from eliminating exposure to cigarette smoke itself. EBV reactivation is the most important risk factor for NPC (50) and is associated with smoking. Several large studies in healthy subjects showed that both smoking (28, 51) and cotinine (52) were associated with higher seropositivity for several bio-markers of EBV reactivation and subsequently with higher risk of NPC (16, 29). Further studies are needed to confirm whether quitting smoking can reduce EBV reactivation, and in turn, prevent the development of NPC.

Major strengths of the present study were the large sample size and detailed information on smoking and quitting, including duration of quitting, age at quitting, and smoking duration and intensity. Moreover, Hong Kong has entered the late stage of the smoking epidemic with a higher proportion of former smokers than other endemic areas in southern China. This, together with its high NPC incidence, has made Hong Kong an opportunistic place for our study.

Our study had several limitations. First, the retrospective study design was subject to recall errors, especially for childhood exposures, although we have shown good reliability of the self-reported lifetime experiences by our previous test-retest study (25). Second, as a case-control study, recall bias, Berkson’s bias, and residual confounding could affect the associations. NPC cases could be more likely to report smoking and quitting than healthy controls who also smoked, but such recall bias would be reduced by using hospital controls who also have diseases. Hospital controls would also improve the similarity of unmeasured characteristics between case and control groups. In addition, indirect Berkson’s bias (overestimated associations because another disease is associated with the exposure under study) would be attenuated, because we used incident cases rather than prevalent cases (53). Third, insufficient women were included largely due to the low smoking and quitting rates, and further studies in women are needed to verify the associations. Ideally, the negative association between quitting and NPC should be confirmed by prospective cohort studies. However, it would be difficult to set up a large cohort and follow them for decades to obtain enough NPC cases who have quit smoking, especially for testing the effects of quitting at young age, quitting for long duration, and dose-response relations. Hence, large case-control studies are probably the most feasible study type for causal inference. Collaborative individual participant data meta-analysis of case-control and cohort studies from all endemic regions are warranted.

## Conclusions

We have shown that longer duration of quitting and younger age at quitting were associated with lower NPC risk with dose-response relations in an NPC endemic region. Our findings support that NPC should be included as a cancer that can be caused by smoking. More stringent tobacco control measures and more effective smoking cessation campaigns and services are needed to prevent NPC.

## Data Availability Statement

The data that support the findings of this study are available from the corresponding authors upon reasonable request.

## Ethics Statement

The studies involving human participants were reviewed and approved by the Institutional Review Board of the HKU/Hospital Authority HK West Cluster (UW 11-192), the HK East Cluster Research Ethics Committee (HKEC-2012-043), the Research Ethics Committee of the Hospital Authority Kowloon Central/Kowloon East (KC/KE-13-0115/ER-2), the Research Ethics Committee of the Kowloon West Cluster [KW/EX-13-073(63-11)], and the NTW Cluster Clinical &amp; Research Ethics Committee (NTWC/CREC/1239-13). The patients/participants provided their written informed consent to participate in this study.

## Author Contributions

SH and T-HL had full access to all the data in the study and take responsibility for the integrity of the data and the accuracy of the data analysis. LW and Z-MM designed the study, collected data, carried out the initial analyses, drafted the initial manuscript, and reviewed and revised the manuscript. RN, W-TN, J-HL, DK, SCC, K-TY, AN, DI, Y-HC, AL, and ML collected data and reviewed and revised the manuscript. SH and T-HL conceptualized the study, supervised the data collection and analyses, and critically reviewed and revised the manuscript. All authors contributed to the article and approved the submitted version.

## Funding

This study was funded by the Hong Kong RGC Area of Excellence Scheme (Grant Number AoE/M-06/08), the World Cancer Research Fund UK (WCRF UK), Wereld Kanker Onderzoek Fonds (WCRF NL), as part of the WCRF International Grant Programme (Grant 2011/460), and Sir Robert Kotewall Professorship in Public Health. The sponsors of the study had no role in study design, data collection, data analysis, data interpretation, or report writing.

## Conflict of Interest

The authors declare that the research was conducted in the absence of any commercial or financial relationships that could be construed as a potential conflict of interest.

## Publisher’s Note

All claims expressed in this article are solely those of the authors and do not necessarily represent those of their affiliated organizations, or those of the publisher, the editors and the reviewers. Any product that may be evaluated in this article, or claim that may be made by its manufacturer, is not guaranteed or endorsed by the publisher.

## References

[1] Chua ML Wee JT Hui EP Chan AT. Nasopharyngeal Carcinoma. Lancet (2016) 387(10022):1012–24. doi: 10.1016/S0140-6736(15)00055-0 26321262

[2] Jia WH Qin HD. Non-Viral Environmental Risk Factors for Nasopharyngeal Carcinoma: A Systematic Review. Semin Cancer Biol (2012) 22(2):117–26. doi: 10.1016/j.semcancer.2012.01.009 22311401

[3] Lee AW Lung ML Ng WT. Nasopharyngeal Carcinoma: From Etiology to Clinical Practice. London, United Kingdom: Academic Press (2019).

[4] Chang ET Adami HO. The Enigmatic Epidemiology of Nasopharyngeal Carcinoma. Cancer Epidemiol Biomarkers Prev (2006) 15(10):1765–77. doi: 10.1158/1055-9965.EPI-06-0353 17035381

[5] Bray F Haugen M Moger TA Tretli S Aalen OO Grotmol T. Age-Incidence Curves of Nasopharyngeal Carcinoma Worldwide: Bimodality in Low-Risk Populations and Aetiologic Implications. Cancer Epidemiol Biomarkers Prev (2008) 17(9):2356–65. doi: 10.1158/1055-9965.EPI-08-0461 18768504

[6] Vaughan TL Stewart PA Teschke K Lynch CF Swanson GM Lyon JL. Occupational Exposure to Formaldehyde and Wood Dust and Nasopharyngeal Carcinoma. Occup Environ Med (2000) 57(6):376–84. doi: 10.1136/oem.57.6.376 PMC1739963 10810126

[7] Liu Z Chang ET Liu Q Cai Y Zhang Z Chen G. Quantification of Familial Risk of Nasopharyngeal Carcinoma in a High-Incidence Area. Cancer (2017) 123(14):2716–25. doi: 10.1002/cncr.30643 PMC5498222 28241094

[8] Xie SH Yu IT Tse LA Au JSK Lau JSM. Occupational Risk Factors for Nasopharyngeal Carcinoma in Hong Kong Chinese: A Case-Referent Study. Int Arch Occup Environ Health (2017) 90(5):443–9. doi: 10.1007/s00420-017-1212-4 28255757

[9] Barrett D Ploner A Chang ET Liu Z Zhang CX Liu Q. Past and Recent Salted Fish and Preserved Food Intakes Are Weakly Associated With Nasopharyngeal Carcinoma Risk in Adults in Southern China. J Nutr (2019) 149(9):1596–605. doi: 10.1093/jn/nxz095 PMC6736189 31127847

[10] IARC Working Group on the Evaluation of Carcinogenic Risks to Humans. Tobacco Smoke and Involuntary Smoking. Lyon (FR: International Agency for Research on Cancer (2004). Available at: https://www.ncbi.nlm.nih.gov/books/NBK316407/. (IARC Monographs on the Evaluation of Carcinogenic Risks to Humans, No. 83.). PMC4781536 15285078

[11] IARC Working Group on the Evaluation of Carcinogenic Risks to Humans. Personal Habits and Indoor Combustions. Lyon (FR: International Agency for Research on Cancer (2012). Available at: https://www.ncbi.nlm.nih.gov/books/NBK304391/. (IARC Monographs on the Evaluation of Carcinogenic Risks to Humans, No. 100E.). PMC4781577 23193840

[12] U.S. Department of Health and Human Services. The Health Consequences of Smoking—50 Years of Progress: A Report of the Surgeon General. Atlanta, GA: U.S. Department of Health and Human Services, Centers for Disease Control and Prevention, National Center for Chronic Disease Prevention and Health Promotion, Office on Smoking and Health (2014).

[13] Xie S-H Yu IT-S Tse LA Au JSK Lau JSM. Tobacco Smoking, Family History, and the Risk of Nasopharyngeal Carcinoma: A Case–Referent Study in Hong Kong Chinese. Cancer Causes Control (2015) 26(6):913–21. doi: 10.1007/s10552-015-0572-x 25822573

[14] Lin JH Jiang CQ Ho SY Zhang WS Mai ZM Xu L. Smoking and Nasopharyngeal Carcinoma Mortality: A Cohort Study of 101,823 Adults in Guangzhou, China. BMC Cancer (2015) 15(1):906. doi: 10.1186/s12885-015-1902-9 26573573 PMC4647498

[15] Chang ET Liu Z Hildesheim A Liu Q Cai Y Zhang Z. Active and Passive Smoking and Risk of Nasopharyngeal Carcinoma: A Population-Based Case-Control Study in Southern China. Am J Epidemiol (2017) 185(12):1272–80. doi: 10.1093/aje/kwx018 PMC5860561 28459936

[16] Hsu WL Chien YC Huang YT Yu KJ Ko JY Lin CY. Cigarette Smoking Increases the Risk of Nasopharyngeal Carcinoma Through the Elevated Level of IgA Antibody Against Epstein-Barr Virus Capsid Antigen: A Mediation Analysis. Cancer Med (2020) 9(5):1867–76. doi: 10.1002/cam4.2832 PMC7050088 31925935

[17] Long M Fu Z Li P Nie Z. Cigarette Smoking and the Risk of Nasopharyngeal Carcinoma: A Meta-Analysis of Epidemiological Studies. BMJ Open (2017) 7(10):e016582. doi: 10.1136/bmjopen-2017-016582 PMC5640018 28982817

[18] Lin JH Wen CP Jiang CQ Yuan JM Chen CJ Ho SY. Smoking and Nasopharyngeal Cancer: Individual Data Meta-Analysis of Six Prospective Studies on 334 935 Men. Int J Epidemiol (2021) 50(3):975–86. doi: 10.1093/ije/dyab060 PMC8271191 33787881

[19] U.S. Department of Health and Human Services. Smoking Cessation. A Report of the Surgeon General. Atlanta, GA: U.S. Department of Health and Human Services, Centers for Disease Control and Prevention, National Center for Chronic Disease Prevention and Health Promotion, Office on Smoking and Health (2020).

[20] Vaughan TL Shapiro JA Burt RD Swanson GM Berwick M Lynch CF. Nasopharyngeal Cancer in a Low-Risk Population: Defining Risk Factors by Histological Type. Cancer Epidemiol Biomarkers Prev (1996) 5(8):587–93. 8824359

[21] Fachiroh J Sangrajrang S Johansson M Renard H Gaborieau V Chabrier A. Tobacco Consumption and Genetic Susceptibility to Nasopharyngeal Carcinoma (NPC) in Thailand. Cancer Causes Control (2012) 23(12):1995–2002. doi: 10.1007/s10552-012-0077-9 23085811

[22] Zhu K Levine RS Brann EA Gnepp DR. Baum MK. A Population-Based Case-Control Study of the Relationship Between Cigarette Smoking and Nasopharyngeal Cancer (United States). Cancer Causes Control (1995) 6(6):507–12. doi: 10.1007/Bf00054158 8580298

[23] Zou J Sun Q Akiba S Yuan Y Zha Y Tao Z. A Case-Control Study of Nasopharyngeal Carcinoma in the High Background Radiation Areas of Yangjiang, China. J Radiat Res (2000) 41 Suppl(Suppl):53–62. doi: 10.1269/jrr.41.s53 11142212

[24] Mai ZM Lin JH Ngan RK Kwong DL Ng WT Ng AW. Milk Consumption Across Life Periods in Relation to Lower Risk of Nasopharyngeal Carcinoma: A Multicentre Case-Control Study. Front Oncol (2019) 9:253. doi: 10.3389/fonc.2019.00253 31024854 PMC6467951

[25] Mai ZM Lin JH Chiang SC Ngan RK Kwong DL Ng WT. Test-Retest Reliability of a Computer-Assisted Self-Administered Questionnaire on Early Life Exposure in a Nasopharyngeal Carcinoma Case-Control Study. Sci Rep (2018) 8(1):7052. doi: 10.1038/s41598-018-25046-y 29728581 PMC5935670

[26] Marmot M Brunner E. Alcohol and Cardiovascular Disease: The Status of the U Shaped Curve. BMJ (Clinical Res ed) (1991) 303(6802):565–8. doi: 10.1136/bmj.303.6802.565 PMC1670912 1912889

[27] GBD 2016 Alcohol Collaborators. Alcohol Use and Burden for 195 Countries and Territories, 1990-2016: A Systematic Analysis for the Global Burden of Disease Study 2016. Lancet (2018) 392(10152):1015–35. doi: 10.1016/s0140-6736(18)31310-2 PMC6148333 30146330

[28] He YQ Xue WQ Xu FH Xu YF Zhang JB Yu HL. The Relationship Between Environmental Factors and the Profile of Epstein-Barr Virus Antibodies in the Lytic and Latent Infection Periods in Healthy Populations From Endemic and Non-Endemic Nasopharyngeal Carcinoma Areas in China. EBioMedicine (2018) 30:184–91. doi: 10.1016/j.ebiom.2018.02.019 PMC5952216 29606628

[29] Xu FH Xiong D Xu YF Cao SM Xue WQ Qin HD. An Epidemiological and Molecular Study of the Relationship Between Smoking, Risk of Nasopharyngeal Carcinoma, and Epstein-Barr Virus Activation. J Natl Cancer Inst (2012) 104(18):1396–410. doi: 10.1093/jnci/djs320 22972969

[30] Marron M Boffetta P Zhang ZF Zaridze D Wunsch-Filho V Winn DM. Cessation of Alcohol Drinking, Tobacco Smoking and the Reversal of Head and Neck Cancer Risk. Int J Epidemiol (2010) 39(1):182–96. doi: 10.1093/ije/dyp291 PMC2817090 19805488

[31] Sriamporn S Vatanasapt V Pisani P Yongchaiyudha S Rungpitarangsri V. Environmental Risk Factors for Nasopharyngeal Carcinoma: A Case-Control Study in Northeastern Thailand. Cancer Epidemiol Biomarkers Prev (1992) 1(5):345–8. 1305465

[32] Yu MC Garabrant DH Huang TB Henderson BE. Occupational and Other Non-Dietary Risk Factors for Nasopharyngeal Carcinoma in Guangzhou, China. Int J Cancer (1990) 45(6):1033–9. doi: 10.1002/ijc.2910450609 2351484

[33] Nam JM McLaughlin JK Blot WJ. Cigarette Smoking, Alcohol, and Nasopharyngeal Carcinoma: A Case-Control Study Among U.S. Whites. J Natl Cancer Inst (1992) 84(8):619–22. doi: 10.1093/jnci/84.8.619 1556772

[34] Chelleng PK Narain K Das HK Chetia M Mahanta J. Risk Factors for Cancer Nasopharynx: A Case-Control Study From Nagaland, India. Natl Med J India (2000) 13(1):6–8. 10743368

[35] Feng BJ Khyatti M Ben-Ayoub W Dahmoul S Ayad M Maachi F. Cannabis, Tobacco and Domestic Fumes Intake Are Associated With Nasopharyngeal Carcinoma in North Africa. Br J Cancer (2009) 101(7):1207–12. doi: 10.1038/sj.bjc.6605281 PMC2768108 19724280

[36] Hsu WL Chen JY Chien YC Liu MY You SL Hsu MM. Independent Effect of EBV and Cigarette Smoking on Nasopharyngeal Carcinoma: A 20-Year Follow-Up Study on 9,622 Males Without Family History in Taiwan. Cancer Epidem Biomar (2009) 18(4):1218–26. doi: 10.1158/1055-9965.Epi-08-1175 19336547

[37] Ji X Zhang W Xie C Wang B Zhang G Zhou F. Nasopharyngeal Carcinoma Risk by Histologic Type in Central China: Impact of Smoking, Alcohol and Family History. Int J Cancer (2011) 129(3):724–32. doi: 10.1002/ijc.25696 20878958

[38] Turkoz FP Celenkoglu G Dogu GG Kalender ME Coskun U Alkis N. Risk Factors of Nasopharyngeal Carcinoma in Turkey-An Epidemiological Survey of the Anatolian Society of Medical Oncology. Asian Pac J Cancer Prev (2011) 12(11):3017–21. 22393983

[39] Lye M-S Visuvanathan S Chong P-P Yap Y-Y Lim C-C Ban E-Z. Homozygous Wildtype of XPD K751Q Polymorphism Is Associated With Increased Risk of Nasopharyngeal Carcinoma in Malaysian Population. PloS One (2015) 10(6):e0130530. doi: 10.1371/journal.pone.0130530 26086338 PMC4472930

[40] Yuan JM Wang XL Xiang YB Gao YT Ross RK Yu MC. Preserved Foods in Relation to Risk of Nasopharyngeal Carcinoma in Shanghai, China. Int J Cancer (2000) 85(3):358–63. doi: 10.1002/(sici)1097-0215(20000201)85:3<358::aid-ijc11>3.0.co;2-e 10652427

[41] Friborg JT Yuan JM Wang RW Koh WP Lee HP Yu MC. A Prospective Study of Tobacco and Alcohol Use as Risk Factors for Pharyngeal Carcinomas in Singapore Chinese. Cancer (2007) 109(6):1183–91. doi: 10.1002/cncr.22501 17315158

[42] Bhatt SP Kim YI Harrington KF Hokanson JE Lutz SM Cho MH. Smoking Duration Alone Provides Stronger Risk Estimates of Chronic Obstructive Pulmonary Disease Than Pack-Years. Thorax (2018) 73(5):414–21. doi: 10.1136/thoraxjnl-2017-210722 PMC5903957 29326298

[43] Li HC Chan SS Lam TH. Smoking Among Hong Kong Chinese Women: Behavior, Attitudes and Experience. BMC Public Health (2015) 15:183. doi: 10.1186/s12889-015-1529-4 25886452 PMC4349309

[44] Yuan JM Wang XL Xiang YB Gao YT Ross RK Yu MC. Non-Dietary Risk Factors for Nasopharyngeal Carcinoma in Shanghai, China. Int J Cancer (2000) 85(3):364–9. doi: 10.1002/(SICI)1097-0215(20000201)85:3<364::AID-IJC12>3.0.CO;2-C 10652428

[45] Lau HY Leung CM Chan YH Lee AW Kwong DL Lung ML. Secular Trends of Salted Fish Consumption and Nasopharyngeal Carcinoma: A Multi-Jurisdiction Ecological Study in 8 Regions From 3 Continents. BMC Cancer (2013) 13:298. doi: 10.1186/1471-2407-13-298 23782497 PMC3729410

[46] Polesel J Franceschi S Talamini R Negri E Barzan L Montella M. Tobacco Smoking, Alcohol Drinking, and the Risk of Different Histological Types of Nasopharyngeal Cancer in a Low-Risk Population. Oral Oncol (2011) 47(6):541–5. doi: 10.1016/j.oraloncology.2011.03.017 21478046

[47] Thurber KA Banks E Joshy G Soga K Marmor A Benton G. Tobacco Smoking and Mortality Among Aboriginal and Torres Strait Islander Adults in Australia. Int J Epidemiol (2021) 50(3):942–54. doi: 10.1093/ije/dyaa274 PMC8271186 33491081

[48] Hashim D Gaughan D Boffetta P Lucchini RG. Baseline Serum Beta-Carotene Concentration and Mortality Among Long-Term Asbestos-Exposed Insulators. Cancer Epidemiol Biomarkers Prev (2015) 24(3):555–60. doi: 10.1158/1055-9965.EPI-14-0952 25542826

[49] Hu T Lin CY Xie SH Chen GH Lu YQ Ling W. Smoking can Increase Nasopharyngeal Carcinoma Risk by Repeatedly Reactivating Epstein-Barr Virus: An Analysis of a Prospective Study in Southern China. Cancer Med (2019) 8(5):2561–71. doi: 10.1002/cam4.2083 PMC6536979 30843658

[50] Young LS Yap LF Murray PG. Epstein–Barr Virus: More Than 50 Years Old and Still Providing Surprises. Nat Rev Cancer (2016) 16(12):789. doi: 10.1038/nrc.2016.92 27687982

[51] He YQ Liao XY Xue WQ Xu YF Xu FH Li FF. Association Between Environmental Factors and Oral Epstein-Barr Virus DNA Loads: A Multicenter Cross-Sectional Study in China. J Infect Dis (2019) 219(3):400–9. doi: 10.1093/infdis/jiy542 PMC6941616 30307559

[52] Yang Q-Y He Y-Q Xue W-Q Zhou T Liao Y Zheng M-Q. Association Between Serum Cotinine Level and Serological Markers of Epstein–Barr Virus in Healthy Subjects in South China Where Nasopharyngeal Carcinoma Is Endemic. Front Oncol (2019) 9:865. doi: 10.3389/fonc.2019.00865 31572673 PMC6753229

[53] Flanders WD Boyle CA Boring JR. Bias Associated With Differential Hospitalization Rates in Incident Case-Control Studies. J Clin Epidemiol (1989) 42(5):395–401. doi: 10.1016/0895-4356(89)90127-3 2732768

